# Toxic epidermal necrolysis induced by moxifloxacin and exacerbated by clindamycin in an elderly patient: A case report

**DOI:** 10.3389/fmed.2025.1527051

**Published:** 2025-02-03

**Authors:** Yongguang Wang, Qizheng Guo, Zhenyang Li, Guifen Gan, Chun Pan

**Affiliations:** ^1^Graduate School of Clinical Medicine, Qinghai University, Xining, China; ^2^Department of Intensive Care Medicine, Affiliated Hospital of Qinghai University, Xining, China; ^3^Department of Intensive Care Unit, Sichuan Provincial People’s Hospital, School of Medicine, University of Electronic Science and Technology of China, Chengdu, China

**Keywords:** toxic epidermal necrolysis, lincosamide antibiotics, corticosteroids, intravenous immunoglobulin, plasma exchange

## Abstract

Toxic Epidermal Necrolysis (TEN) is a severe skin-mucosal reaction induced by medications, commonly characterized by blister formation and widespread epidermal detachment. Typical causative agents include allopurinol, antibiotics, anticonvulsants, and non-steroidal anti-inflammatory drugs (NSAIDs), with trimethoprim/sulfamethoxazole and penicillin being the most frequently implicated among antibiotics. However, lincosamide antibiotics as the cause of aggravation are rarely reported in clinical settings. This case report presents a patient with TEN induced by quinolone antibiotics, who experienced rapid progression of the condition after combining with lincomycin antibiotics. Clinical remission was achieved through a combination of corticosteroids, intravenous immunoglobulin, and plasma exchange therapy. This report aims to enhance clinicians’ understanding of TEN by providing a detailed case presentation and discussion.

## Introduction

Toxic Epidermal Necrolysis (TEN) is a rare and severe cutaneous adverse drug reaction, characterized by widespread epidermal detachment, and is induced by a Type IV hypersensitivity reaction (1). It is typically triggered by medications and is known for its rarity and high mortality rate (2). The mortality rate of TEN is closely associated with increasing age. Advanced age is not only a risk factor for the occurrence of TEN but also a factor linked to increased mortality (3, 4). Common drugs that trigger TEN include allopurinol, antibiotics, anticonvulsants, and non-steroidal anti-inflammatory drugs (NSAIDs). Studies indicate that drugs with a high risk of inducing TEN include allopurinol, carbamazepine, lamotrigine, sulfonamide antibiotics, and sulfasalazine, while drugs with moderate risk include cephalosporins, quinolones, macrolides, and tetracyclines (5). Currently, there are no definitive diagnostic criteria or standardized clinical treatment guidelines for TEN. In addition to discontinuing the causative drugs and providing symptomatic supportive care, common systemic therapies include corticosteroids, intravenous immunoglobulin (IVIG), etanercept, and plasma exchange. This case report describes a patient with severe TEN, triggered by the quinolone antibiotic moxifloxacin and potentially exacerbated by the lincosamide antibiotic clindamycin. The patient’s condition improved following treatment with corticosteroids, intravenous immunoglobulin, and plasma exchange. Lincosamide antibiotics as a trigger for TEN are rare, and the therapeutic effect of the combination of corticosteroids, intravenous immunoglobulin, and plasma exchange remains unclear (6). Therefore, this case report may provide valuable insights into drug screening for the diagnosis of TEN and offer a reference for the prognosis of TEN treated with corticosteroids, intravenous immunoglobulin, and plasma exchange.

## Case presentation

**FIGURE 1:**
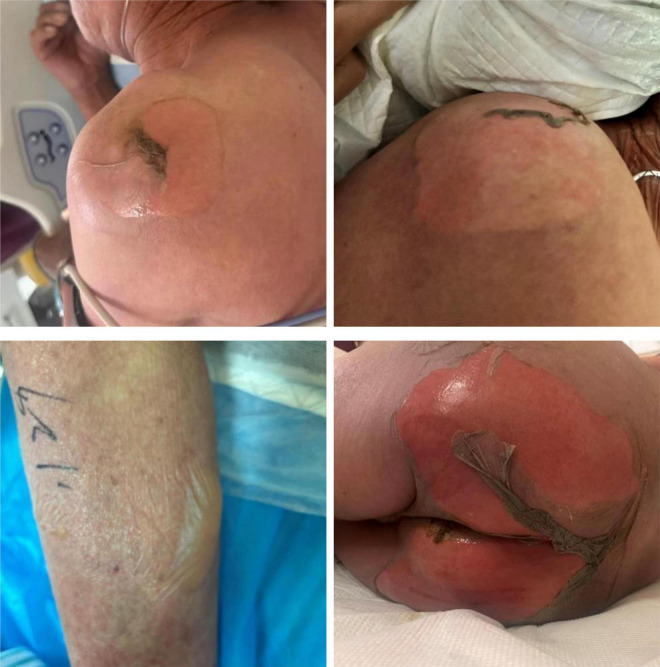
Multiple areas of ulceration on the patient’s shoulders, buttocks, and lower limbs on the day of admission, with new blister formation on the lower limbs.

An 81-year-old male patient from China presented with symptoms of fever, cough, sputum production, and shortness of breath one week before admission. The highest recorded temperature during the fever was 41°C. He was treated with moxifloxacin at a local hospital, but his wheezing symptoms showed no significant improvement. Additionally, he developed pruritus, and in the days leading up to his visit to the emergency department, he experienced mild skin ulceration and erosion accompanied by pain. He also had frequent episodes of epistaxis and recurrent oral ulcers. Upon visiting the emergency department, the patient was treated with clindamycin, dexamethasone, and aminophylline. Two hours after initiating treatment, the patient’s pruritus and pain significantly worsened. Skin ulcerations and pain on his shoulders, buttocks, and lower limbs became more severe, and new blisters developed on his left ankle and right lower limb within a short period (Figure 1). Physical examination revealed diffuse erythema throughout his body, with large blisters on the shoulders, buttocks, and limbs, particularly concentrated around the left ankle and scattered over the right lower limb. Some of the blister walls had ruptured and eroded. A positive Nikolsky sign was observed, with the total affected area exceeding 30% (Figure 1). Moderate edema was noted in both lower limbs. In addition, varying sizes of ulcers and blisters were observed on the nasal and oral mucosa. The patient was admitted to the intensive care unit for further treatment.

The patient’s medical history included a 50-year history of tuberculosis, 15 years of benign prostatic hyperplasia, chronic bronchitis, and allergies to penicillin, cephalosporin antibiotics, Shenmai injection (a traditional Chinese medicine injection), and coriander. Upon admission, routine blood tests showed the following results: red blood cell count: 3.83 × 10^12/L (normal range: 4.30–5.80), white blood cell count: 10.28 × 10^9/L (normal range: 3.50–9.50), neutrophil count: 6.53 × 10^9/L (normal range: 1.8–6.3). Serum IgG was 6.3 g/L (normal range: 7–16) and complement C3 was 0.638 g/L (normal range: 0.88–1.8). Other tests, including ANA antibodies, γ-interferon release assay, rheumatoid factor (RF), anti-streptolysin O (ASO), anti-cyclic citrullinated peptide IgG (AntiCCP IgG), cytoplasmic anti-neutrophil cytoplasmic antibody (cANCA), perinuclear anti-neutrophil cytoplasmic antibody (pANCA), anticardiolipin antibodies, and HIV antibodies, all returned negative results. Additionally, the Human Leukocyte Antigen B27 (HLA-B27) test was also negative.

TEN does not have a standardized diagnostic criterion, but certain features are suggestive, including macular targetoid lesions, involvement of two mucous membranes, recent drug exposure, and histopathological findings. TEN can be further differentiated from Stevens-Johnson Syndrome (SJS) based on the extent of skin involvement. Typically, in SJS, the affected body surface area is less than 10%; in SJS-TEN overlap, the affected area ranges from 10 to 30%; while in TEN, the total skin involvement exceeds 30%, representing the most severe drug-induced skin reaction (7, 8). In this reported case, the patient developed fever, cough, sputum production, and shortness of breath one week prior to admission. Following drug treatment, the patient exhibited generalized pruritus and skin ulceration, which progressively worsened with an affected area greater than 30%. Therefore, based on the clinical history and physical examination findings, the diagnosis of TEN was considered.

Clinically, many other diseases can present with similar skin rashes. For example, drug-induced bullous fixed drug eruption, characterized by recurrent rashes at the same site after each exposure to the causative drug, along with excessive post-inflammatory hyperpigmentation lasting for weeks to months, was excluded in this case as the patient did not meet these characteristics (9). Staphylococcal Scalded Skin Syndrome (SSSS) typically starts with edematous erythema and fissured erythema, followed by blister formation, with crusting and radiating fissures around the eyes and mouth. However, the rash characteristics in this case did not align with those of SSSS, so this diagnosis was also excluded (10). Erythema Multiforme (EM), often triggered by herpes simplex virus, typically presents with target lesions on the extremities, and the lesions are irregular red macules (11). Laboratory testing for herpes simplex virus was negative, and the lesions did not meet the criteria for EM, making this diagnosis unlikely as well.

**FIGURE 2:**
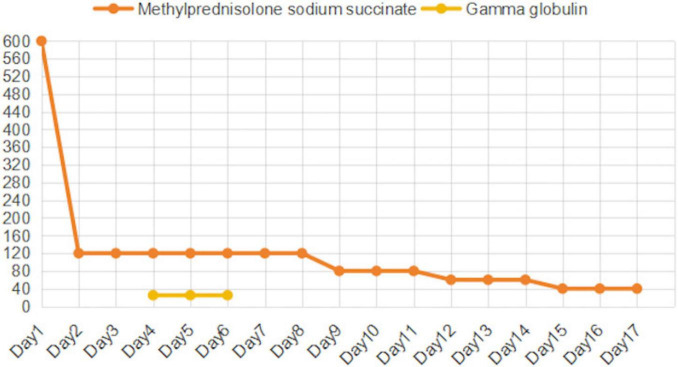
Changes in Methylprednisolone (mg/d) and Intravenous Immunoglobulin (g/d) Doses.

The treatment of TEN currently lacks standardized clinical guidelines and requires an individualized treatment plan based on the patient’s actual condition and disease progression. Upon admission, the causative drugs (moxifloxacin and clindamycin) were immediately discontinued, and high-dose methylprednisolone (600 mg) was initiated as shock therapy (Figure 2), with the dose tapered to 120 mg/day on the second day. The patient also presented with multiple ulcers on the lips and oral mucosa, which were treated with 0.9% saline and nystatin mouthwash. After two days, the condition of the oral and lip mucosa improved. Epistaxis caused by nasal mucosal involvement was not treated specifically but resolved spontaneously with ongoing TEN treatment.

Despite 4 days of corticosteroid treatment, pruritus, skin ulceration, and erosion showed no improvement, and new blisters developed on the lower limbs. Epidermal growth factor and fusidic acid ointment were applied to the affected areas, and exudates were aspirated from the blisters. To further control disease progression, corticosteroids were combined with IVIG and plasma exchange. IVIG (25 g/day) and the first plasma exchange (2740 mL) were initiated on the same day.

By day 6, pruritus had improved, and no new blisters formed, though ulceration and erosion persisted. A second plasma exchange (2,600 mL) was performed, while corticosteroids were maintained at 120 mg/day alongside IVIG. On day 7, due to financial constraints, the family refused further IVIG after 3 days of therapy. Despite this, the patient’s condition improved, with no new blisters and stabilization of skin lesions. On day 9, a third plasma exchange (1,990 mL) was performed, and the methylprednisolone dosage was reduced to 80 mg/day.

By day 12, partial healing of erosions was observed, with drying and desquamation of rashes on the lower limbs. The corticosteroid dose was tapered to 60 mg/day. By day 15, ulcerated areas had scabbed, and methylprednisolone was further reduced to 40 mg/day. After 17 days of hospitalization, the patient was discharged with instructions to continue methylprednisolone (20 mg/day) and attend follow-up appointments.

One-month post-discharge, the patient reported full recovery, with skin completely healed and cessation of all medications three weeks after discharge.

## Discussion

The patient in this case is an 81-year-old male with more than 30% of his body surface affected, and with underlying conditions including tuberculosis, chronic bronchitis, and benign prostatic hyperplasia, placing him in a critical condition with a high mortality rate. TEN is closely related to adverse drug reactions, with its pathogenesis strongly associated with drug-induced immune-mediated reactions (8). A retrospective descriptive study (12) found that antibiotics, anticonvulsants, and NSAIDs are the three most common drug classes that cause TEN, with causality rates of 56.7, 23.3, and 16.7%, respectively. Among antibiotics, the most common causative drugs were trimethoprim/sulfamethoxazole, with a causality rate of 41.1%. In this study, we used the Naranjo Score Criterion (13) and the ALDEN scoring criteria (14) to evaluate moxifloxacin and clindamycin, respectively. The ALDEN scoring system is specifically designed for SJS/TEN and is considered more sensitive and reliable than the Naranjo Score Criterion in assessing the causal relationship between suspected drugs and SJS/TEN. The Naranjo Score Criterion assigned a score of 3 to moxifloxacin and 2 to clindamycin, indicating that both drugs are possible causes, though moxifloxacin is slightly more likely to be the primary culprit clinically (Table 1). The ALDEN scoring criteria assigned 5 points to moxifloxacin and 2 points to clindamycin, suggesting that moxifloxacin is more likely to be the primary cause in this case, while clindamycin may have contributed to the worsening of the condition (Table 2). However, due to the patient’s financial constraints, relevant genotypic studies could not be completed, so clindamycin cannot be completely ruled out.

**TABLE 1:** Naranjo score criterion and comparison of naranjo score between moxifloxacin and clindamycin.

Question	Yes	No	Do not know	Moxifloxacin	Clindamycin
1. Are there previous conclusive reports on this reaction?	+1	0	0	Yes (+1)	No (0)
2. Did the adverse event appear after the suspected drug was administered?	+2	−1	0	Yes (+2)	Yes (+2)
3. Did the adverse reaction improve when the drug was discontinued or a specific antagonist was administered?	+1	0	0	Yes (0)	Yes (0)
4. Did the adverse reaction reappear when the drug was readministered?	+2	−1	0	Do not know (0)	Do not know (0)
5. Are there alternative causes (other than the drug) that could on their own have caused the reaction?	−1	+2	0	Do not know (0)	Do not know (0)
6. Did the reaction reappear when a placebo was given?	−1	+1	0	Do not know (0)	Do not know (0)
7. Was the drug detected in the blood (or other fluids) in concentrations known to be toxic?	+1	0	0	Do not know (0)	Do not know (0)
8. Was the reaction more severe when the dose was increased, or less severe when the dose was decreased?	+1	0	0	Do not know (0)	Do not know (0)
9. Did the patient have a similar reaction to the same or similar drugs in any previous exposure?	+1	0	0	No (0)	No (0)
10. Was the adverse event confirmed by any objective evidence?	+1	0	0	No (0)	No (0)

The results of the Naranjo scoring system are divided into four levels: Impossible (≤0), Possible (1–4), Probable (5–8), Definite (≥9).

**TABLE 2:** ALDEN scoring criteria and comparison of ALDEN score between moxifloxacin and clindamycin.

Criterion	Values	Rules to apply	Moxifloxacin	Clindamycin
Delay from initial drug intake to onset of reaction	Suggestive (+3), Compatible (+2), Likely (+1), Unlikely (-1), Excluded (-3)	5 to 28 days (+3), 29 to 56 days (+2), 1 to 4 days (+1), > 56 days (-1), started on or after index day (-3)	+3 (Suggestive)	+1(Likely)
Drug present in the body on index day	Definite (0), Doubtful (-1), Excluded (-3)	Continued to index day ( < 5 times elimination half-life), stopped > 5 times with/without liver/kidney alterations	0 (Definite)	0 (Definite)
Prechallenge/rechallenge	Positive specific (+4, +2), Positive unspecific (+1), Not done/unknown (0), Negative (-2)	SJS/TEN after same drug (+4), similar drug (+2), other reactions (+1), no reaction or unknown (-2, 0)	0 (Not done/unknown)	0 (Not done/unknown)
Dechallenge	Neutral (0), Negative (-2)	Stopped (0), continued without harm (-2)	0 (Neutral)	0 (Neutral)
Type of drug (notoriety)	Strongly associated (+3), Associated (+2), Suspected (+1), Unknown (0), Not suspected (-1)	High-risk drug (+3), lower risk drug (+2), ambiguous evidence (+1), unknown/new drugs (0), no evidence (-1)	+3 (Strongly associated)	+3 (Strongly associated)
Other cause	Possible (-1)	If another drug has an intermediate score > 3, subtract 1 from others	−1 (Possible)	−1 (Possible)
Final score	−12 to 10	< 0: Very unlikely, 0–1: Unlikely, 2–3: Possible, 4–5: Probable, > 6: Very probable	+5 (Probable)	+5 (Probable)

<0, Very unlikely; 0–1, unlikely; 2–3, possible; 4–5, probable; ≥6, very probable. ATC, anatomical therapeutic chemical; SJS, Stevens-Johnson syndrome; TEN, toxic epidermal necrolysis.

Currently, there are no standardized treatment guidelines for TEN, but the first step in treatment is the discontinuation of the causative drugs and all non-essential medications, which plays a crucial role. This should be followed by systemic therapy. In existing studies, early high-dose corticosteroid therapy has been shown to improve patient outcomes (15). Intravenous immunoglobulin (IVIG) has also been shown to play a role in treating TEN by inhibiting Fas receptor-mediated apoptosis. A network meta-analysis showed that combined corticosteroid and IVIG therapy can reduce mortality in TEN patients. However, studies suggest that the therapeutic window for corticosteroids and IVIG is quite narrow, with better outcomes only if administered within 1–2 days after skin damage appears (16). There is also a potential risk of increased skin infections and renal failure, which limits the use of IVIG alone. Therefore, the current recommendation is for the use of corticosteroids alone, or in combination with IVIG, but not IVIG alone (17, 18). Severe cutaneous adverse reactions (SCARs) mediated by cytotoxic T lymphocytes, including SJS and TEN, have been shown to be effectively treated with anti-TNF-α biologic agents, and Etanercept is one of anti-TNF-α biologic agents (19). Etanercept has also been proven to be an effective treatment with minimal side effects. Administering etanercept twice a week until healing is the only method that has shown low-certainty evidence of effectiveness (20–22), and it has been found to reduce mortality more effectively than corticosteroids (6). When used in combination with corticosteroids, it may provide greater benefit for TEN patients. A multicenter retrospective study showed that etanercept in combination with corticosteroids, or with corticosteroids and IVIG, resulted in a more significant improvement in mortality (23).

In addition to pharmacological therapy, serum purification plays a crucial role in TEN management. The most effective method for serum purification in TEN patients currently is the combination of corticosteroids and plasma exchange. A cohort study involving 59 TEN patients found that those receiving high-dose corticosteroids combined with plasma exchange were all cured. In this case, the patient was treated with an individualized approach using corticosteroids, intravenous immunoglobulin, and plasma exchange (24). As a result, the patient’s skin ulceration improved, and the skin lesions on the shoulders, buttocks, and limbs healed completely, providing further evidence for the effectiveness of the combination therapy.

We employed the blister aspiration technique, a traditional Chinese nursing method, during treatment. Currently, there is no consensus on whether blisters caused by TEN should be aspirated. In this case, we regularly aspirated fluid from blisters larger than 1 cm on the patient’s lower limbs. While blister formation does not directly cause epidermal detachment, it softens and moistens the epidermis, increasing the risk of rupture and subsequent separation. Additionally, friction between the blisters and bed linens during movement or repositioning could lead to rupture, exposing the damaged skin to multi-drug-resistant bacteria in the ward environment or direct contact with bed linens, potentially causing local or systemic infections and worsening the prognosis. Regular aspiration of subepidermal blisters was therefore considered beneficial for maintaining epidermal integrity, preventing infections, and reducing complications from ruptures in the ICU.

In conclusion, TEN is a severe, drug-induced, skin-mucosal-dominant systemic immune-mediated reaction with no standardized clinical treatment guidelines. In addition to early discontinuation of the causative drugs, clinical treatment should be individualized based on the patient’s condition. Currently, effective treatments include corticosteroids combined with IVIG and/or plasma exchange, and etanercept combined with corticosteroids. Future studies should aim to conduct more comprehensive basic and clinical research, establish standardized treatment guidelines, and optimize treatment protocols to improve patient outcomes.

## Data Availability

The original contributions presented in the study are included in the article/supplementary material, further inquiries can be directed to the corresponding author.
